# Synergy between Sphingosine 1-Phosphate and Lipopolysaccharide Signaling Promotes an Inflammatory, Angiogenic and Osteogenic Response in Human Aortic Valve Interstitial Cells

**DOI:** 10.1371/journal.pone.0109081

**Published:** 2014-10-02

**Authors:** Isabel Fernández-Pisonero, Javier López, Esther Onecha, Ana I. Dueñas, Patricia Maeso, Mariano Sánchez Crespo, José Alberto San Román, Carmen García-Rodríguez

**Affiliations:** 1 Instituto de Biología y Genética Molecular (CSIC-Universidad Valladolid), Valladolid, Spain; 2 Cardiology Department, Instituto Ciencias del Corazón (ICICOR) Hospital Clínico Universitario, Valladolid, Spain; 3 Research Unit, Hospital Clínico Universitario, Valladolid, Spain; Faculty of Medicine & Health Sciences, United Arab Emirates

## Abstract

Given that the bioactive lipid sphingosine 1-phosphate is involved in cardiovascular pathophysiology, and since lipid accumulation and inflammation are hallmarks of calcific aortic stenosis, the role of sphingosine 1-phosphate on the pro-inflammatory/pro-osteogenic pathways in human interstitial cells from aortic and pulmonary valves was investigated. Real-time PCR showed sphingosine 1-phosphate receptor expression in aortic valve interstitial cells. Exposure of cells to sphingosine 1-phosphate induced pro-inflammatory responses characterized by interleukin-6, interleukin-8, and cyclooxygenase-2 up-regulations, as observed by ELISA and Western blot. Strikingly, cell treatment with sphingosine 1-phosphate plus lipopolysaccharide resulted in the synergistic induction of cyclooxygenase-2, and intercellular adhesion molecule 1, as well as the secretion of prostaglandin E_2_, the soluble form of the intercellular adhesion molecule 1, and the pro-angiogenic factor vascular endothelial growth factor-A. Remarkably, the synergistic effect was significantly higher in aortic valve interstitial cells from stenotic than control valves, and was drastically lower in cells from pulmonary valves, which rarely undergo stenosis. siRNA and pharmacological analysis revealed the involvement of sphingosine 1-phosphate receptors 1/3 and Toll-like receptor-4, and downstream signaling through p38/MAPK, protein kinase C, and NF-κB. As regards pro-osteogenic pathways, sphingosine 1-phosphate induced calcium deposition and the expression of the calcification markers bone morphogenetic protein-2 and alkaline phosphatase, and enhanced the effect of lipopolysaccharide, an effect that was partially blocked by inhibition of sphingosine 1-phosphate receptors 3/2 signaling. In conclusion, the interplay between sphingosine 1-phosphate receptors and Toll-like receptor 4 signaling leads to a cooperative up-regulation of inflammatory, angiogenic, and osteogenic pathways in aortic valve interstitial cells that seems relevant to the pathogenesis of aortic stenosis and may allow the inception of new therapeutic approaches.

## Introduction

Calcific aortic stenosis is the most frequent cause of aortic valve replacement in developed countries [1]. The underlying disease-driving mechanisms are not fully understood, although the role of inflammation, lipid accumulation, matrix remodeling, angiogenesis, and the renin-angiotensin system has been demonstrated [1], [2], [3], [4]. After clinical trials showing no significant effects of lipid lowering statins [5], invasive valve replacement or transcatheter aortic valve implantation are the only effective therapies [4], [6].

Sphingosine 1-phosphate (S1P), a bioactive lipid mediator synthesized by platelets, endothelial cells and erythrocytes [7], [8], regulates various cellular functions, including proliferation, survival, migration, adhesion, and inflammation [8], and plays a role in the cardiovascular system [9], [10]. S1P is mainly associated to lipoproteins and albumin and its concentrations are around µM in plasma and nM in tissues. S1P can either act as an intracellular second messenger or on the cell surface in an autocrine or paracrine manner by binding to G protein-coupled receptors known as S1P_1-5_, which generate multiple signals and a fine-tuning of specific responses [8], [11]. S1P receptors are widely expressed in the cardiovascular system, where divergent roles have been reported, including pro- and anti-atherogenic effects [9], [10], cardioprotection [10], [12], [13], and cardiac fibrosis [14].

Toll-like receptors (TLRs) are innate immune receptors involved in the detection of molecular patterns present in pathogens and endogenous molecules released upon cell damage and necrosis [15]. Increasing evidence has shown the involvement of TLRs in the homeostasis and the pathology of the cardiovascular system [16], [17], mainly regarding TLR4, the receptor for the lypopolysaccharide (LPS) present in Gram-negative bacteria, and TLR2, the sensor for bacterial lipoproteins and lipoteichoic acid [15]. Recent reports have demonstrated a connection between TLRs and aortic stenosis, as TLR2/4/3 activation promote pro-inflammatory and pro-osteogenic responses in human aortic valve interstitial cells (AVIC) [18], [19].

Given the prominence of lipid accumulation and inflammatory changes in aortic stenosis, and S1P involvement in cardiovascular pathophysiology, the role of S1P in the pro-inflammatory/pro-osteogenic responses was investigated in AVIC from stenotic and non-stenotic valves, and compared to valve interstitial cells from pulmonary valves (PVIC). Our data demonstrate a synergy between S1P and LPS at a p38 MAPK-dependent signaling step that enhances pro-inflammatory and pro-osteogenic events in interstitial cells from the aortic valve and may be relevant to the pathogenesis of the disease.

## Materials and Methods

### Ethics Statement

The Review Board from the Hospital Clínico Universitario de Valladolid approved the study, which complies with the Declaration of Helsinki. All patients gave written informed consent prior to surgery, following a procedure approved by the Ethics Committee from the Hospital.

### Cell Isolation, Culture, and Characterization

The study included 15 explanted heart valves from patients with degenerative severe aortic stenosis (11 males/4 females, 74±7 years). Aortic valve area was 0.7±0.2 cm^2^, peak gradient 78±19 mmHg and mean gradient 55±13 mmHg. In addition, 15 aortic valves and 15 pulmonary valves from transplant recipients with valve disease excluded by echocardiography (12 males/3 females, 59±10 years) were studied. Diagnosis and indications for valve replacement and heart transplantation were performed following European guidelines. Interstitial cells from aortic and pulmonary valves were isolated using sequential collagenase digestion, characterized with α-SM-actin staining, and cultured as described [18], [19], [20]. Three types of cultured interstitial cells were investigated, namely stenotic AVIC (from stenotic aortic valve), control AVIC (from non-stenotic aortic valve), and control PVIC (from non-stenotic pulmonary valve). In culture, more than 90% of stenotic AVIC, control AVIC, and control PVIC stained positively for α-SM-actin, consistent with a myofibroblast phenotype in the three cell types used for the study (Figure S1).

### Real-time RT-PCR Analysis

First-strand cDNA was synthesized from total RNA by the reverse transcription reaction, and later amplified by PCR using primer sequences for human S1P receptors, as described [19], [21]. β-actin was used as a housekeeping gene to assess the relative abundance of mRNA. Quantification of the mRNA levels was performed by using the Delta Delta Ct method, where Ct is the cycle threshold value. The Ct of the sample was normalized to the Ct of β-actin, and later normalized to the value of the sample with the lowest expression.

### Cytokine Expression Analysis by Antibody Arrays and ELISA

Cells were stimulated for 12 h with the indicated ligand, S1P or LPS from *E. coli* type 0111:B4 (Sigma, St. Louis, MO). Supernatants were analyzed with Human Cytokine Antibody Array 5 (RayBiotech, Norcross, GA), as described [19]. Cytokines analyzed include: ENA-78, G-CSF, GM-CSF, GRO, GRO alpha, I-309, IL-1 alpha, IL-1 beta, IL-2, IL-3, IL-4, IL-5, IL-6, IL-7, IL-8, IL-10, IL-12 p40/p70, IL-13, IL-15, IFN-gamma, MCP-1, MCP-2, MCP-3, M-CSF, MDC, MIG, MIP-1 beta, MIP-1 delta, RANTES, SCF, SDF-1, TARC, TGF beta 1, TNF alpha, TNF beta, EGF, IGF-1, Angiogenin, Oncostatin M, Thrombopoietin, VEGF-A, PDGF-BB, Leptin, BDNF, BLC, Ck beta 8-1, Eotaxin-1, Eotaxin-2, Eotaxin-3, FGF-4, FGF-6, FGF-7, FGF-9, Flt-3 ligand, Fractalkine, GCP-2, GDNF, HGF, IGFBP-1, IGFBP-2, IGFBP-3, IGFBP-4, IL-16, IP-10, LIF, Light, MCP-4, MIF, MIP-3 alpha, NAP-2, NT-3, NT-4, Osteopontin, Osteoprotegerin, PARC, PLGF, TGF beta 2, TGF beta 3, TIMP-1, and TIMP-2. Secretion of IL-6, IL-8, PGE_2_, the soluble form of the intercellular adhesion molecule 1 (ICAM-1) and vascular endothelial growth factor (VEGF)-A was evaluated by immunoassay kits following the manufacturer's protocol (GE-Healthcare, Buckinghamshire, UK; RayBiotech, Norcross, GA). Absorbance was measured using a microplate reader Versamax (Molecular Devices, Sunnyvale, CA).

### Immunoblotting for the Detection of Pro-Inflammatory and Pro-Osteogenic Molecules

Cells were activated with S1P and/or LPS for the indicated times. Lysates were analyzed by Western blot using antibodies against human cyclooxygenase-2 (COX-2) and ICAM-1, and the phosphorylated forms of NF-κB-p65 and MAPK. An anti-β-tubulin antibody was used as a load control, as described [19]. Bone morphogenetic protein (BMP)-2 detection was performed as reported [18]. In pharmacological studies, cells were pre-treated for 30 min with either S1P receptors antagonists W146 (Cayman Chem., Ann Arbor, MI); VPC 23019 (Avanti Polar Lipids, Alabaster, AL); JTE-013 (Tocris, Bristol, UK); suramin (Biomol, Santa Fe, NM), or TLR4 signaling antagonists CLI-095 (InvivoGen, San Diego, CA); CAY10614 (Cayman Chem., Ann Arbor, MI) or signaling cascades inhibitors NF-κB SN50, ALLN, SB203580, and GF109203X (Calbiochem, Darmstadt, Germany); PD98059 (Tocris, Bristol, UK); pertussis toxin (PTX) and SP600125 (Sigma, St. Louis, MO).

### siRNA Interference

Cells were transfected with a liposome-base reagent Dharmafect (Dharmacon, Lafayette, CO) following manufacturer's guidelines as described [18]. Briefly, 100 nM of siRNA, resuspended in Opti-MEM, were mixed with the Dharmafect reagent to obtain RNA-liposome conjugates, and later incubated with cells for 24 h. siRNA duplexes for S1P receptor silencing were the validated siRNA duplexes specific for S1P human receptors, and a negative silencer RNA control (Ambion, Austin, TX): S1P_1_ (#4143, #145848), S1P_2_ (#45076, #44984), and S1P_3_ (#1959, #1875). Real-time PCR was performed to confirm S1P receptors knock-down after 24 h of transfection, as described above. The degree of inhibition rated from 50-90% for S1P_1_, 50% for S1P_2_, and 70% for S1P_3_. Transfected cells were activated and ICAM-1 and COX-2 were analyzed by Western blot.

### *In Vitro* Calcification, and Quantification of Calcium Deposition and Alkaline Phosphatase (ALP) Activity

For calcification experiments, cells were cultured in conditioning medium (M199 supplemented with 10 mmol/l β-glycerophosphate, 10 nmol/l vitamin D_3_, and 10 nmol/l dexamethasone), as described [19], and stimulated with the indicated agonists or vehicle every three days. Inhibitors were incubated 1h before the stimulation. For the quantification of calcium deposition, cells were stimulated for 25-27 days, and later decalcified with 0.6M HCl for 24 h. The calcium levels were determined using a calcium colorimetric assay Kit (Biovision Inc., Milpitas, CA) based on the o-cresolphtalein complexone method, as described [22]. To evaluate ALP expression, after 17-19 days of stimulation, cells were fixed with p-formaldehyde, stained with the ALP blue kit as previously described [19]. For measuring ALP activity, cells were lysed and the enzymatic activity was evaluated using a fluorometric assay kit (Abcam, Cambridge, UK), as described [19].

### Statistical Analysis

To analyze the effect of one factor (i.e. treatment), data were analyzed by a One way-ANOVA with the Tukey posthoc test using GraphPad Prism version 6 (San Diego, CA); in the case of one factor and 2 levels, a t test was performed. To analyze the effect of two factors (i.e. cell x treatment, time x treatment; cell x treatment at time 10 min), data were analyzed by Two-way ANOVA with the LSD Fisher posthoc test. To analyze the effect of three factors (cell x treatment x time), data were analyzed by a Three-way ANOVA using Statgraphics Centurion XVI 16.2.04 (StatPoint Technologies, Inc. USA; Warrenton, VA). Statistic analysis is detailed in Table S1. Differences were considered statistically significant for a p<0.05.

## Results

### S1P Induces Pro-Inflammatory Molecule Expression in AVICs

**Figure 1:**
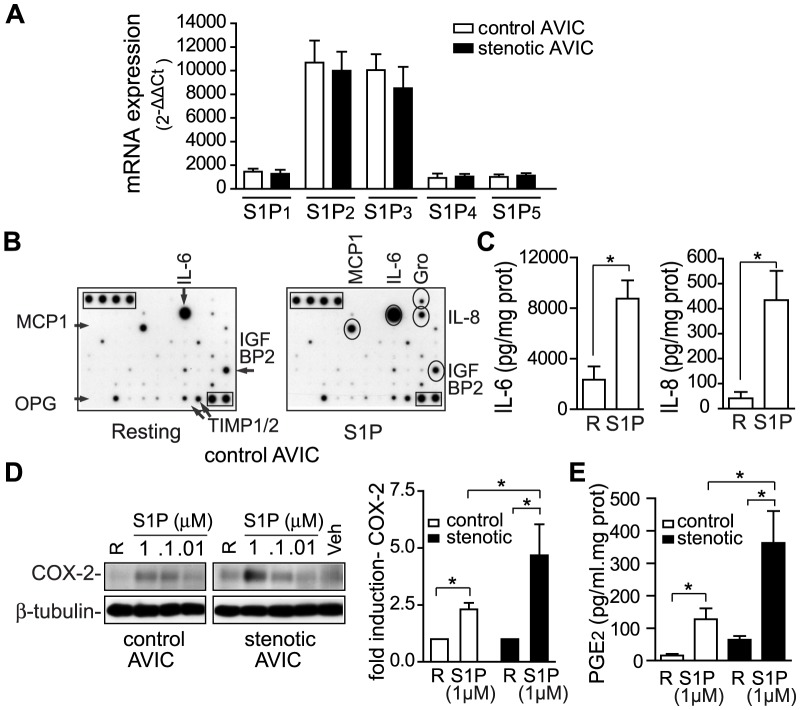
S1P induces pro-inflammatory molecules in AVICs. A) S1P receptor expression in control and stenotic AVICs (mean ± SEM of the relative mRNA levels normalized to β-actin, n = 7–10), was assessed by quantitative RT-PCR. B–C) 1 µM S1P induced cytokine secretion, as observed in antibody arrays and ELISA assays (representative of n = 4, 12 h). Squares indicate positive controls; arrows, constitutive cytokines; ovals, S1P-induced cytokines. D–E) Comparison of S1P-mediated induction of COX-2 expression (mean ± SEM, n = 8–12, 1 µM S1P) and PGE_2_ secretion (mean ± SEM, n = 4–5). ELISA data, expressed as pg, were normalized to the cell protein content (mg). *p<0.05. White bars indicate control AVIC; black bars, stenotic AVIC. Gro indicates growth-regulated oncogene α, β, and γ; MCP-1, monocyte chemotactic protein-1; R, resting.

Quantitative RT-PCR experiments revealed S1P receptor expression in AVIC, being S1P_2/3_ the most abundant ones (Figure 1A). No differences at the mRNA level were observed between cells from control and stenotic valves (Figure 1A). Exposure of control AVIC to S1P promoted the secretion of several pro-inflammatory cytokines (i.e. IL-6, IL-8, Gro, monocyte chemoattractant protein (MCP)-1), as observed in a cytokine antibody array (Figure 1B), and quantified by ELISA (Figure 1C). Additionally, a dose-dependent induction of COX-2 by S1P was observed by Western blot analysis (Figure 1D). Interestingly, the up-regulation of COX-2 and of its predominant product PGE_2_ were more prominent in stenotic than in control AVICs (Figures 1D-E), thus suggesting that AVIC from stenotic valves display a lasting enhanced capacity to produce lipid inflammatory mediators in response to S1P. Together, these data show a S1P-mediated pro-inflammatory phenotype in AVIC.

### Synergy between S1P and LPS Promotes a Marked Pro-Inflammatory and Pro-Angiogenic Phenotype in AVIC, with a More Significant Effect in Stenotic AVIC

**Figure 2:**
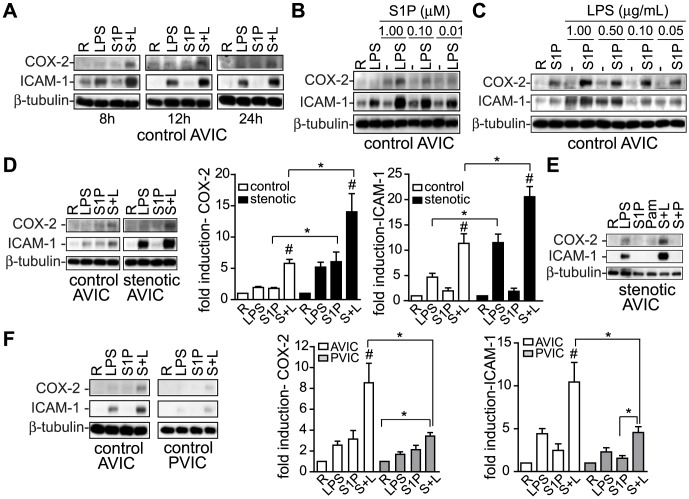
S1P cooperates with LPS to up-regulate pro-inflammatory molecules. A) Representative immunoblots show the kinetics of COX-2 and ICAM-1 induction upon activation in control AVIC (n = 8). B–C) Dose-dependent effect (n = 3, 12 h). D) Immunoblots with densitometry data normalized to β-tubulin levels (mean ± SEM, n = 10 pairs of control-stenotic AVICs activated for 12 h and processed in the same blot) revealed a stronger synergistic effect in stenotic than in control AVICs. E) Representative immunoblots of at least 5 experiments show no cooperative effect between S1P and Pam_3_CSK4 in stenotic AVICs. F) Immunoblots with densitometry data (mean ± SEM, n = 8 pairs of AVIC-PVIC from the same heart processed in the same blot), demonstrate a higher effect in AVIC than in PVIC. White bars indicate control AVIC; black bars, stenotic AVIC; gray bars, PVIC. L indicates 1 µg/ml LPS; Pam/P, 100 ng/ml Pam_3_CSK4; S, 1 µM S1P. #*p*<0.05 for S1P+LPS vs. S1P and LPS;*p<0.05 for the indicated pair.

In light of a previous study demonstrating TLR2-S1P receptors negative crosstalk in human macrophages [21], we sought to investigate the potential interaction of S1P and LPS. In control AVICs, S1P and LPS induced the expression of pro-inflammatory molecules such as COX-2 and the adhesion molecule ICAM-1 after 8–24 h (Figure 2A). Strikingly, cell exposure to S1P+LPS induced a remarkable up-regulation of COX-2 and ICAM-1 expression (Figure 2A). The cooperative effect was dose-dependent and observed in the range 1–0.01 µM of S1P (Figure 2B) and 1–0.1 µg/ml of LPS (Figure 2C). The effect showed the features of a synergistic cooperation between S1P and LPS, because it was higher than the sum of the effect of either ligand (Figure 2D). Strikingly, the cooperative effect on COX-2 and ICAM-1 up-regulation was statistically significantly higher in AVICs from stenotic than control valves (Figure 2D). Conversely, treatment with S1P plus the TLR2/TLR1 ligand Pam_3_CSK4 showed no synergistic induction of COX-2 and ICAM-1 (Figure 2E), consistent with the low TLR2 expression reported in AVICs [18], [19], and arguing for a TLR4-specific effect. Interestingly, when comparing AVIC and PVIC isolated from the same patient, the up-regulation of COX-2 and ICAM-1 was significantly higher in cells from aortic than from pulmonary valves (Figure 2F), which rarely have stenosis and have a lower TLR4 expression [18].

**Figure 3:**
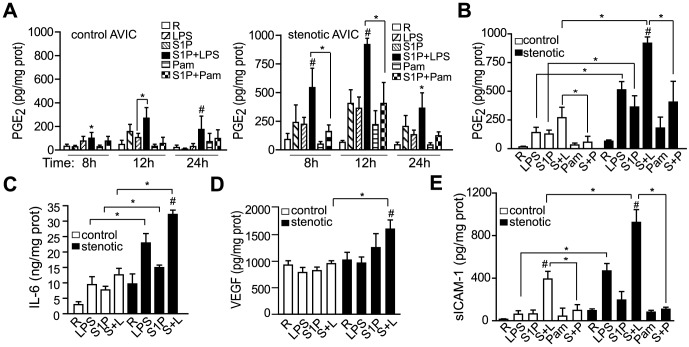
S1P cooperates with LPS to induce the secretion of pro-inflammatory and pro-angiogenic molecules. Supernatants from cells treated with the indicated ligands as in Figure 2 were analyzed by ELISA. Data are expressed as pg/mg cell protein (mean ± SEM). A) Kinetics of PGE_2_ secretion in control and stenotic AVIC, n = 4–5. B) PGE_2_ secretion data from A at 12 h, mean ± SEM, n = 4–5. C) IL-6 secretion data at 12 h, representative of 4 independent experiments. D) VEGF secretion data at 12 h, mean ± SEM, n = 6. E) sICAM-1 secretion data, mean ± SEM, n = 5–10.) Abbreviations were as in Figure 2; color bars, as indicated in the corresponding panel. *p<0.05; #*p*<0.05 for S1P+LPS vs. LPS and S1P.

In agreement with COX-2 up-regulation, S1P+LPS, but not S1P+Pam_3_CSK4, cooperated to induce PGE_2_ secretion in AVICs (Figure 3A), being the effect statistically significantly higher in cells from stenotic than from control valves (Figures 3A–B). Additionally, S1P cooperated with LPS to increase IL-6 secretion, being the induction statistically significantly higher in stenotic than in control AVICs (Figure 3C). Since the presence of the angiogenic mediator VEGF-A has been reported in stenotic aortic valves [3], [23] and angiogenesis is known to be co-dependent with chronic inflammation in several diseases [24], the induction of VEGF-A was explored. Interestingly, S1P, known to induce angiogenesis, cooperated with LPS to promote a statistically significant secretion of VEGF-A by stenotic AVIC, while no significant effects were observed in control AVIC (Figure 3D). Altogether, data suggest that S1P and LPS cooperate to induce a marked pro-inflammatory and pro-angiogenic phenotype in human AVICs, with a more significant effect in cells from stenotic valves and lower in cells from pulmonary valves.

### S1P and LPS Cooperate to Induce the Secretion of the Soluble Form of ICAM-1 (sICAM-1)

sICAM-1, which has been associated with the severity and prevalence of the calcification in the aortic valve disease in the Multi-Ethnic Study of Atherosclerosis (MESA) [25], was evaluated by ELISA. sICAM-1 was detected in the supernatants of LPS-activated AVICs, and its levels increased synergistically in the presence of S1P (Figure 3E), thus agreeing with ICAM-1 up-regulation (Figure 2D). Moreover, the effect was statistically significantly higher in control than in stenotic AVIC (Figure 3E). Conversely, treatment with S1P+Pam_3_CSK4 showed no synergistic induction of sICAM-1 in stenotic AVIC (Figure 3E), arguing for a TLR4-specific effect. Together, the data demonstrate that S1P exacerbates LPS-mediated release of the calcification biomarker sICAM-1 by AVICs.

### S1P Receptors and Pro-Inflammatory Routes Involved in the Synergistic Effect with TLR4

**Figure 4:**
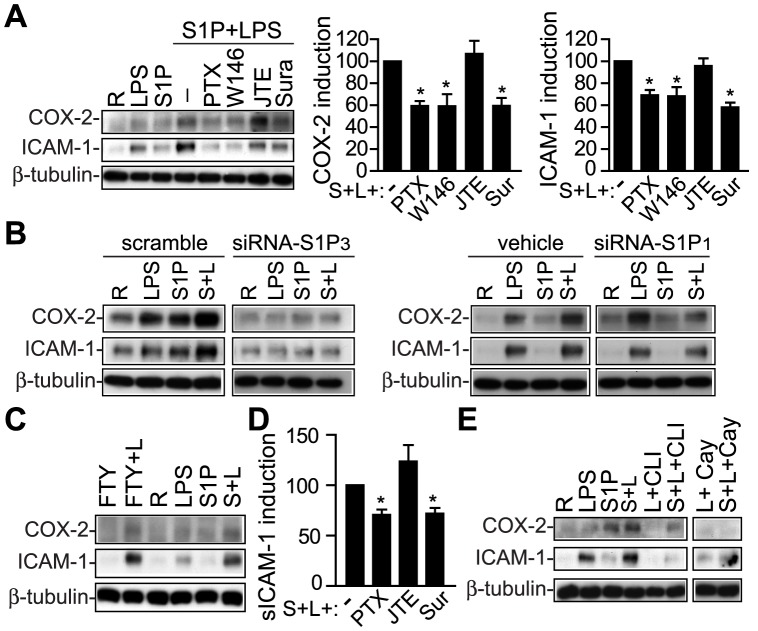
Receptors involved in the cooperative effect. AVICs were pre-treated with the indicated drugs, activated for 12 h, and cell lysates and supernatants were analyzed by Western blot and ELISA, respectively. A) Representative immunoblots with densitometry data demonstrate inhibition of the cooperative effect on COX-2 and ICAM-1 expression (100% value) using S1P_1/3_ antagonists (n = 6–12). B) Silencing S1P_1/3_ attenuated the cooperative effect (n = 3 control AVIC). Scramble, siRNA control; vehicle, 0.1% DEPC. C) Immunoblots showed cooperation between FTY720 and LPS (n = 3). D) ELISA quantification of sICAM-1 levels show inhibition by S1P_1/3_ antagonists (n = 6–10). E) Immunoblots demonstrate inhibition of the cooperative effect by TLR4 antagonists (n = 3). Cay indicates 5 µM CAY10614; CLI, 3 µM CLI-095; FTY, 1 µM FTY720; JTE, 10 µM JTE-013; S+L, S1P+LPS; PTX, 100 ng/ml *pertussis* toxin; R, resting; Sur, 10 µM suramin; W146, 10 µM W146. *p<0.05 vs. S1P+LPS.

Synergistic effects between S1P and LPS on COX-2 and ICAM-1 up-regulation were inhibited by pre-treatment with suramin, a S1P_3_ antagonist, W146, a S1P_1_ antagonist, PTX, which blocks S1P_1-3_ signaling (Figure 4A), and by knocking down S1P_1/3_ expression using a siRNA technique (Figure S2 and Figure 4B), but not by the S1P_2_ antagonist JTE-013 (Figure 4A). Synergy with LPS was mimicked by FTY720, a S1P analogue that binds to all S1P receptors but S1P_2_ (Figure 4C). Additionally, the synergistic effect on sICAM-1 was also sensitive to PTX and suramin (Figure 4D). Moreover, COX-2 and ICAM-1 up-regulation was abrogated by blocking the LPS/TLR4 route with CAY10614 and CLI-095, respectively (Figure 4E).

**Figure 5:**
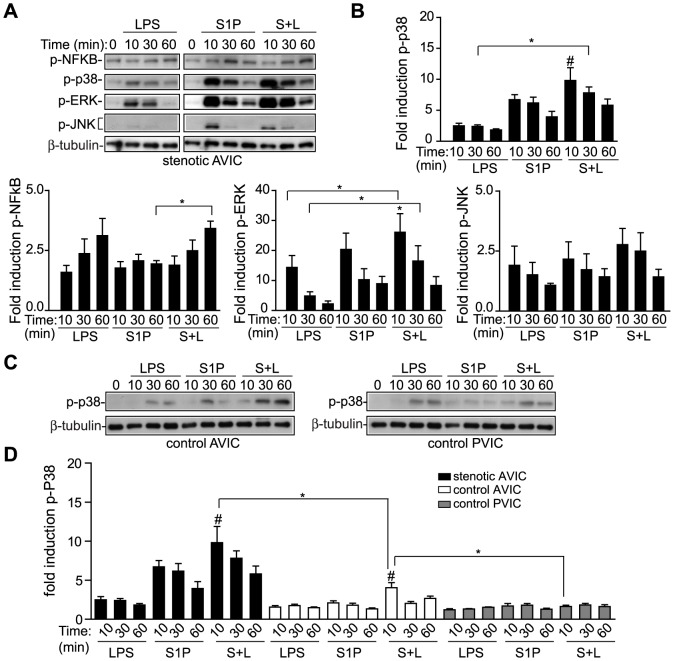
Several signaling cascades, mainly p38/MAPK, are involved in the cooperative effect. A–B) Cell lysates from activated stenotic AVIC were analyzed for the early phosphorylation of NF-κB and MAP kinases. Representative immunoblots and densitometry data show synergistic activation of p38 by S1P+LPS (n = 6). Sample at t = 0 was run in both gels for comparison purposes. C) Representative immunoblots of p38 phosphorylation in AVIC and PVIC from the same patient processed in parallel are shown. D) Densitometry data is expressed as the fold induction of p-p38 relative to resting values (t = 0) using data previously normalized to the reference gene, β-tubulin (mean ± SEM, n = 5–7). Color bars, as in Figure 2. *p<0.05; #*p*<0.05 for S1P+LPS vs. LPS and S1P (at the same time point).

The analysis of intracellular signaling revealed that AVIC exposure to S1P+LPS leads to the early activation of NF-κB and MAPK routes (Figures 5A–B). Interestingly, treatment with S1P+LPS induced the phosphorylation of p38, but not NF-κB, ERK, or JNK, in a synergistic manner, since p38 phosphorylation was higher that the obtained by the sum of the effect of each ligand alone (Figures 5A–B), thus suggesting that the p38/MAPK pathway might be a cross-road signaling point. When the effect on p38 activation was tested in control AVIC and PVIC from the same patient, control AVIC, but not PVIC, showed a cooperation effect with S1P+LPS (Figure 5C). In order to compare data from the different cell types, data was expressed as fold induction of p38 phosphorylated as compared to t = 0 (Figure 5D). As shown in Figure 5D, the cooperation effect of S1P+LPS on p38 activation was statistically higher in stenotic than in control valve cells and, in addition the induction was lower in cells from pulmonary than aortic valves (Figure 5D). Differences might account for their different inflammatory responsiveness.

**Figure 6:**
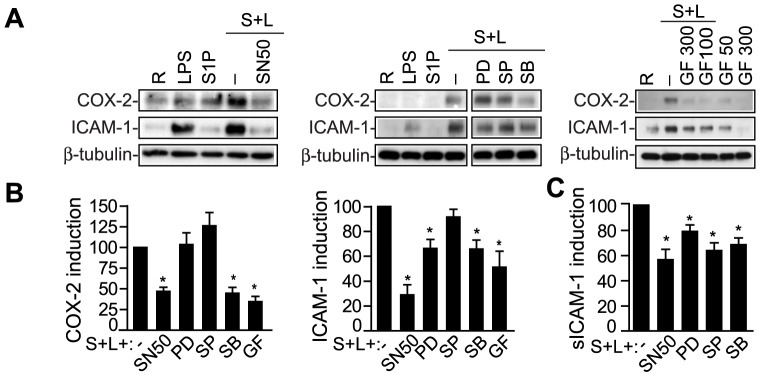
Signaling routes implicated in the cooperative effect. A–B) AVIC were pre-treated with the indicated drugs, activated for 12 h, and analyzed as in Figure 4. Representative immunoblots of AVIC lysates and densitometry data show inhibition of the cooperative effect on COX-2 and ICAM-1 (100% value) by NF-κB-SN50 and MAPK inhibitors (n = 8). C) Supernatants were analyzed for sICAM-1 as in Figure 3 (n = 6). GF indicates 300 nM GF109203X; PD, 50 µM PD98059; SB, 10 µM SB203580; S+L, S1P+LPS; SN50; 50 µg/mL NF-κB SN50; SP, 10 µM SP600125. *p<0.05 vs. S1P+LPS (100% value).

As to the induction of pro-inflammatory molecules, and consistent with the signaling analysis data (Figures 5A–B), the p38/MAPK inhibitor SB203580 blocked the synergistic up-regulation of COX-2 and ICAM-1 (Figures 6A–B) as well as sICAM-1 secretion (Figure 6C). The synergistic effect on COX-2 and ICAM-1 was inhibited by the PKC antagonist GF109203X (Figures 6A-B), which agrees with the involvement of this kinase upstream of p38 MAPK. In addition, NF-κB-SN50, which prevents activation of the pro-inflammatory gene regulator NF-κB, inhibited the S1P+LPS-mediated up-regulation of COX-2 and ICAM-1 (Figures 6A–B) and sICAM-1 secretion (Figure 6C), thus indicating a role of the NF-κB route. The effect on ICAM-1 and sICAM-1 induction was also significantly reduced by the MEK inhibitor PD98059 (Figures 6A–B). Altogether, these results demonstrate the involvement of several S1P receptors and pro-inflammatory cascades, mainly involving p38/MAPK, in the synergistic effects with TLR4.

### S1P and LPS Cooperate to Induce the Pro-Osteogenic Markers BMP-2 and ALP as well as Calcium Deposition

**Figure 7:**
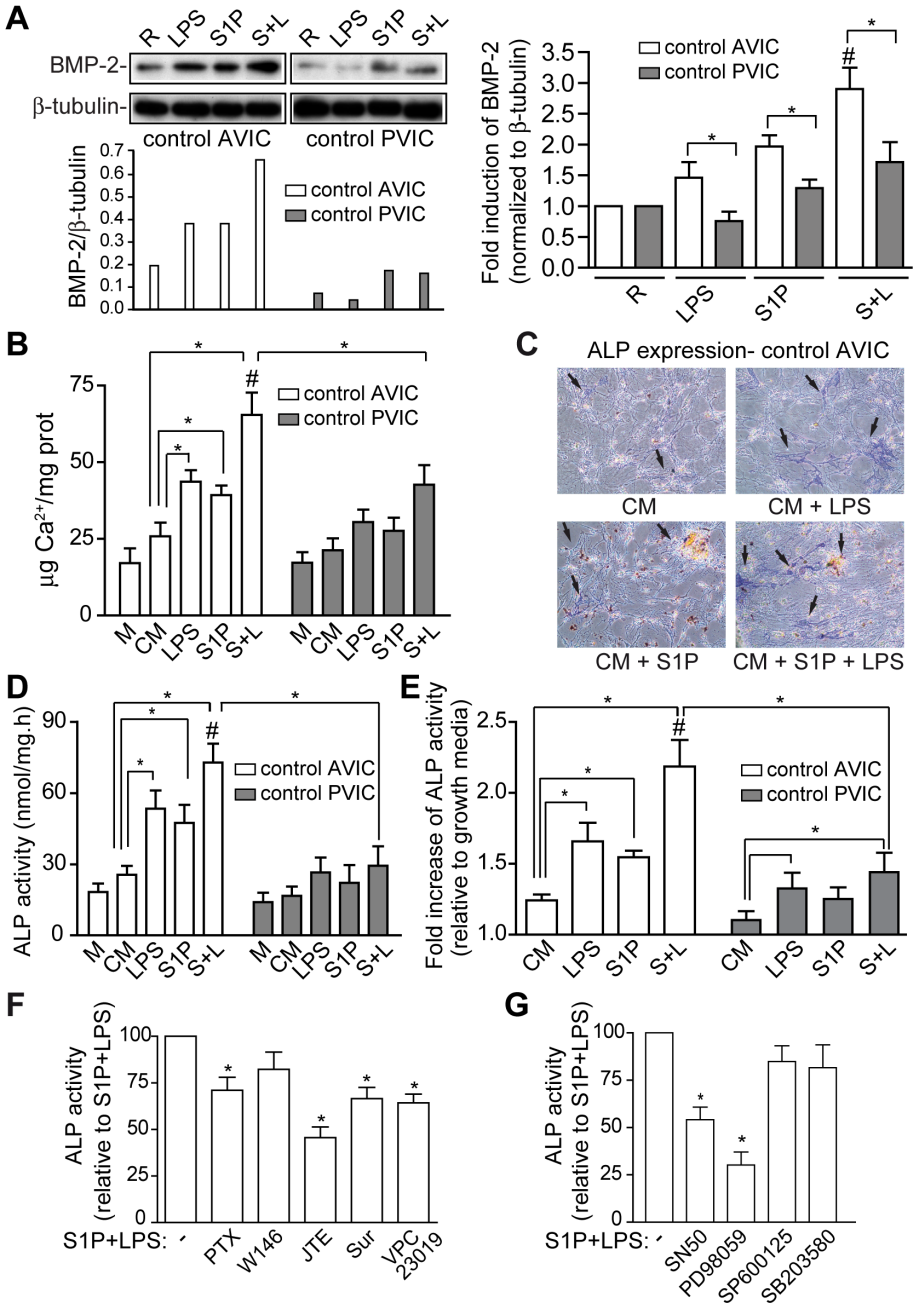
S1P cooperates with LPS to up-regulate pro-osteogenic markers and calcium deposition. A) AVIC and PVIC from the same patient were activated as in Figure 2 for 48 h and analyzed with a BMP-2 antibody. Immunoblots and densitometry data are representative of at least 3 pairs of AVIC-PVIC processed in the same blot. Data are expressed as fold induction of BMP2, normalized to β-tubulin, as compared to resting conditions (mean ± SEM, n = 6 pairs of AVIC-PVIC). B–G) AVIC and PVIC from the same patient were treated with conditioning media (CM) in the presence of 1 µg/ml LPS, 0.1 µM S1P or vehicle as indicated in Methods. B) Calcium deposition expressed as µg Ca^2+^/mg protein is shown (mean ± SEM, n = 3 pairs of AVIC and PVIC with a total of 11 replicates). C) ALP blue staining of control AVIC (n = 3). D) Representative ALP data is expressed as nmol/mg.h. E) Data from control AVIC and PVIC are expressed as fold increase of ALP activity (mean ± SEM, n = 12 AVIC, n = 8 PVIC) relative to data with growth media. F–G). ALP activity in control AVIC pre-treated with the indicated drugs before activation (n = 5–9) Drug concentrations were as in Figures 4–5. M indicates M199, growth media; white bars, AVIC; gray bars, PVIC. *p<0.05; #*p*<0.05 for S1P+LPS vs. LPS and S1P.

Western blot analysis revealed the S1P-mediated induction of BMP-2 in control AVICs (Figure 7A), being the effect comparable to that elicited by LPS, known to up-regulate BMP-2 in AVIC [18]. Moreover, S1P-mediated induction was higher in AVIC than PVIC from the same patient (Figure 7A). Furthermore, S1P cooperated with LPS to further increase BMP-2 expression in AVIC (Figure 7A).

In *in vitro* calcification studies S1P induced calcium deposition, and cooperated with LPS to further increase calcium deposits in control AVIC (Figure 7B). In addition, S1P induced cell aggregation and ALP expression in control AVIC (Figure 7C), an effect which was similar to that elicited by LPS. Moreover, S1P+LPS further increased ALP expression (Figure 7C) and ALP activity (Figure 7D–E) in control AVIC. Additionally, calcium content (Figure 7B) and ALP activity up-regulation (Figures 7D–E) were significantly lower in PVICs than in AVICs isolated from the same patient. The cooperative effect on ALP activity was significantly inhibited by the S1P_2_ antagonist JTE-013 and to a lesser extent by the S1P_3_ antagonist suramin, by VPC23019, a S1P_1/3_ antagonist, and was PTX-sensitive (Figure 7F), thus suggesting the involvement of S1P_2/3_ subtype receptors. The cooperative effect was also inhibited by NF-κB-SN50 and the MEK inhibitor PD98059 (Figure 7G), thus indicating the involvement of the NF-κB and ERK/MAPK routes. Together, these data demonstrate that S1P induces *in vitro* calcification and intensifies the LPS-induced pro-osteogenic phenotype in AVICs.

## Discussion

The present data disclose the role of S1P on the induction of inflammation and osteogenesis in human AVICs, and support the relevance of a two-signal paradigm leading to the induction of enhanced responses, because S1P increases the activity of the LPS/TLR4 route. Since the synergistic effects are significantly higher in AVICs from stenotic than control valves and lower in PVIC, and can be blocked with S1P receptor and TLR4 antagonists, the S1P receptors-TLR4 interplay might have potential long-term pathophysiologically relevant consequences and offers new molecular targets for aortic stenosis treatment.

Aortic valves might be exposed to S1P originated from blood and vascular cell sources, i.e. endothelial cells activated by physiological fluid shear-stress, platelet activation/aggregation occurring in active cardiovascular disease states, and erythrocytes [7], [8]. Also, S1P is associated with lipoproteins, which are present in stenotic aortic valves [2]. Our study shows that AVIC mainly express S1P_2-3_, as compared to predominant subtypes S1P_1-3_ in the heart [10], making it likely that, the distinct cell-dependent expression could account for differential response to S1P. Additionally, our data shows that S1P up-regulates several chemokines involved in the recruitment of inflammatory cells like IL-8, Gro, and MCP-1, and cytokines like IL-6, being the cytokine profile similar to the described for LPS in a previous study [19]. Moreover, S1P induces COX-2 expression and PGE_2_ secretion in AVICs, arguing for a potential role of eicosanoids in the pathogenesis of aortic stenosis. Consistent with this concept, recent data have shown the up-regulation of the 5-lipoxygenase pathway in human aortic valves with severe stenosis, and the potentially detrimental role of leukotrienes on valvular myofibroblasts [26].

These results show that S1P promotes aggregation and calcification of human AVICs and agree with the reported role of S1P on osteoimmunology by osteoclast precursor mobilization and bone homeostasis [27], and with a recent report describing S1P-mediated contraction and nodule formation in porcine AVICs [28]. Additionally, oxidized LDL, which contain S1P [9], [7], has been proposed to have a potential role in the development of calcific aortic valve disease [29]. Moreover, sphingosine kinase 1 overexpression has been shown to promote cardiomyocyte degeneration and fibrosis *in vivo* [14]. On the other hand, the S1P/sphingosine kinase axis has a role on cardioprotection after ischemic injury in cardiac myocytes and *ex vivo* murine hearts by acting as an endogenous cardioprotectant released by ischemic pre- and post-conditioning [13].The divergent roles of S1P on cardiovascular pathophysiology might be explained by the different subtypes of S1P receptor expressed and by the distinct signaling routes involved.

An interesting finding outlined in this report is that S1P exacerbates the LPS/TLR4-induced inflammatory/osteogenic phenotype in AVICs. This synergistic induction may have pathophysiological relevance given the stronger effect observed in cells from stenotic as compared to control valves, and in cells from aortic versus pulmonary valves. In AVIC, integration of TLR and S1P signaling pathways might dictate the magnitude of the inflammatory response and contribute to disease. Based on our data, we propose that a two-signal paradigm best explains a synergistic inflammatory response leading to calcification in the valvular disease. On this basis, two signals might be required to activate a robust inflammatory response in human AVICs: i) exposure to microbial products and/or endogenous ligands originated by cell damage and necrosis and ii) stimulation by S1P released from platelet or endothelial or erythrocytes. Our findings are reminiscent of recent reports demonstrating S1P receptor-TLR4 cooperation to induce cytokine production in human gingival epithelial cells [30], and cytokine/adhesion molecule expression in human endothelial cells [31]. Another report shows that TLR4 and Notch1 pathways crosstalk increases the inflammatory response in stenotic AVIC [32]. An important aspect is the cell-specificity of the TLRs-S1P receptor crosstalk. In stark contrast, S1P_1/2_ negatively regulates TLR2-signaling in human monocytes/macrophages and this could explain some S1P-mediated anti-atherogenic properties [21], thus stressing of its dependence on the cell context and the microenvironment.

Interestingly, S1P and LPS cooperate to induce the secretion of the pro-angiogenic factor VEGF-A in stenotic but not in control AVIC. These results are consistent with a recent report proposing that mast cells and myofibroblasts may promote valvular neovascularisation by modifying the angiogenic/anti-angiogenic factor balance [33]. Additionally, the differences between control and stenotic AVICs are consistent with the reported faster formation of angiogenic sprouts in stenotic than in control valves [34]. Moreover, we observed that S1P and LPS cooperate to induce the secretion of the inflammatory mediators PGE_2_ and IL-6, which have been reported to induce VEGF [35]. In addition, IL-6 has been reported to induce endothelial cell migration [36], a crucial step in angiogenesis.

Cooperation of S1P and LPS on the induction of the pro-osteogenic and calcification biomarkers supports the concept of inflammation-dependent development of calcific aortic valve disease emerging from *in vitro*, clinical studies, and multimodal molecular imaging studies [3], [37], [26]. Calcification is less frequent in pulmonary than in aortic valves and a mechanical hypothesis has been proposed to explain those differences in pathology, since the pulmonary valve leaflets are under a significantly less severe mechanical stress than aortic valve leaflets [38]. In favor of an alternative hypothesis is a report emphasizing that the TLR-mediated pro-inflammatory and pro-osteogenic phenotype in AVIC is not observed in PVIC [18]. Consistent with a valve-specific response, the magnitude of the S1P receptor-TLR4 cooperative effect in PVIC is significantly lower than in AVIC from the same patient, which provides a molecular explanation of why stenosis is rarely observed in pulmonary valves. AVICs have the machinery to fine-tuning inflammatory responses that may become inappropriate on the face of repeated pro-inflammatory stimuli, since cells from diseased valves showed a more robust synergistic effect. A question raised by these findings is how inappropriate responses could lead to osteoblastic differentiation.

In light of our findings, therapeutic interference with S1P receptor and TLR4 signaling could be a potentially useful strategy to slow down aortic stenosis progression by avoiding inappropriate inflammatory responses. Our data demonstrate the involvement of S1P_3/1_ in inflammation and S1P_2/3_ in calcification responses in interplay with TLR4. Recently, it has been proposed that S1P_3_ plays a causal role in atherosclerosis by promoting monocyte/macrophage recruitment and altering smooth muscle cell behavior [39], and it seems involved in cardiac myofibroblast differentiation [40]. S1P_2_ has been associated with regeneration and fibrosis after liver [41], and S1P_1_ has been linked to pulmonary fibrosis [42].

The molecular basis of cooperative induction of inflammatory molecules by S1P and LPS seems to rely on an intersection on the p38/MAPK signaling route. The different levels of induction of p38 activation might account for the differences in the pro-inflammatory and pro-osteogenic responses observed in stenotic versus control AVIC, and in aortic versus pulmonary valve cells. The p38/MAPK signaling route has been implicated in cardiac hypertrophy, ischemic injury, and heart failure [43]. Moreover, data demonstrate that PKC, an upstream kinase that activates TAK1 and the p38 kinase activator MKK3, seems to be involved in the effect. The TAK1-MKK3/6-p38MAPK signaling axis has been reported to be important for TGF-beta-related cardiac hypertrophy [44]. Furthermore, our data demonstrate the involvement of NF-κB, in particular the p50/p65 heterodimers, in the S1P receptor-TLR4 cooperative effect on inflammation/osteogenesis. Interestingly, whereas the p38/MAPK seems implicated in the synergistic induction of pro-inflammatory molecules, the ERK/MAPK is involved in the cooperative effect on osteogenesis, consistent with its reported role in AVIC calcification [45].

In conclusion, these data disclose S1P as a novel inducer of inflammation and calcification of human AVIC. Moreover, the pair S1P receptors-TLR4 may be potential targets for aortic stenosis treatment since S1P cooperates with LPS to induce synergistic inflammatory, angiogenic, and osteogenic responses.

## Supporting Information

Figure S1 **Characterization of AVICs from stenotic and control aortic valves and PVIC from control pulmonary valves.** Cells were labeled with a FITC-conjugated antibody against α-SM-actin followed by DAPI staining to visualize nuclei, and later analyzed by fluorescence microcopy (Nikon ECLIPSE 90i fluorescence microscope coupled to a Nikon DS-Ri1 camera). Representative images of cells correspond to stenotic (left panel) and control aortic valve cells (central panel), and to control pulmonary valve cells (right panel), being control AVIC and control PVIC isolated from the same patient. Images were obtained using Image J software. In green are shown images from FITC staining (positively labeled with α-SM-actin); in blue are images from DAPI staining (nuclei). More than 95% cells in culture were myofibroblast. No apparent morphological differences in cells explanted from control and stenotic valves were observed.
(TIF)

Figure S2 **Knock-down of S1P receptor mRNA expression by siRNA technique.** Cells were transfected with RNA duplex that inhibited mRNA expression of the indicated S1P receptor genes, or a siRNA control (scramble) or the vehicle. Plots represent the quantitative PCR profiles in comparison with the housekeeper gene β-actin. Images are representative of 3 independent experiments.
(EPS)

Table S1 **Summary of Statistical Analysis.** Detailed information on the statistics corresponding to the indicated figures is shown.
(DOC)
