# Supplementary figures and images for: Synergy between Sphingosine 1-Phosphate and Lipopolysaccharide Signaling Promotes an Inflammatory, Angiogenic and Osteogenic Response in Human Aortic Valve Interstitial Cells
Source: PLoS One. 2014 Oct 2;9(10):e109081. doi: 10.1371/journal.pone.0109081 (PMC4183546; doi:10.1371/journal.pone.0109081)

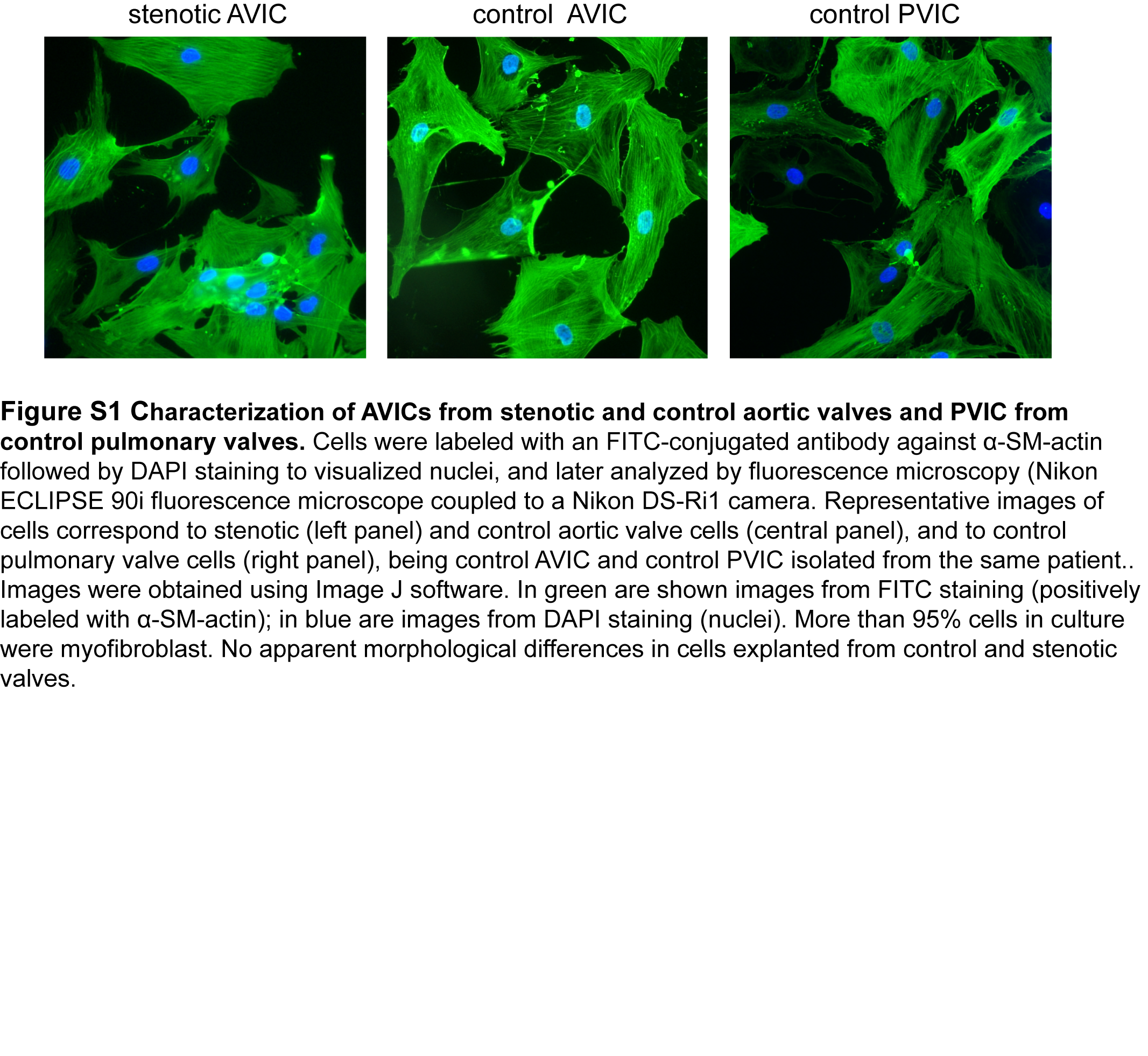

Supplement: Figure S1 — Characterization of AVICs from stenotic and control aortic valves and PVIC from control pulmonary valves. Cells were labeled with a FITC-conjugated antibody against α-SM-actin followed by DAPI staining to visualize nuclei, and later analyzed by fluorescence microcopy (Nikon ECLIPSE 90i fluorescence microscope coupled to a Nikon DS-Ri1 camera). Representative images of cells correspond to stenotic (left panel) and control aortic valve cells (central panel), and to control pulmonary valve cells (right panel), being control AVIC and control PVIC isolated from the same patient. Images were obtained using Image J software. In green are shown images from FITC staining (positively labeled with α-SM-actin); in blue are images from DAPI staining (nuclei). More than 95% cells in culture were myofibroblast. No apparent morphological differences in cells explanted from control and stenotic valves were observed. (TIF) [file pone.0109081.s001.tif]
