# Supplementary material for: Synergy between Sphingosine 1-Phosphate and Lipopolysaccharide Signaling Promotes an Inflammatory, Angiogenic and Osteogenic Response in Human Aortic Valve Interstitial Cells
Source: PLoS One. 2014 Oct 2;9(10):e109081. doi: 10.1371/journal.pone.0109081 (PMC4183546; doi:10.1371/journal.pone.0109081)
Supplement: Table S1 — Summary of Statistical Analysis. Detailed information on the statistics corresponding to the indicated figures is shown. (DOC) [file pone.0109081.s003.doc]

**STATISTICAL ANALYSIS**

**FIGURE 1**

**(A)** S1P receptor expression. Two-way ANOVA (cell x S1P receptor) revealed a significant main effect of S1P receptor (F _(4, 63)_ = 30.18, *P* < 0.0001) and non significant effect of cell (F _(1, 63)_ = 27.81, *P* = 0.5918) and a non significant interaction between factors (F _(4, 63)_ = 0.1491, *P* = 0.9627).

**(C)** IL-6 and IL-8 data.

- IL-6 data. A t test revealed a significant effect (*P* = 0.027)

- IL-8 data. A t test revealed a significant effect (*P* = 0.0168)

**(D)** COX2 data for stenotic vs control AVIC. Two-way ANOVA (cell x treatment) revealed a significant main effect of cell (F _(1, 27)_ = 7.46, *P* = 0.0110) and a significant effect of treatment (F _(1, 27_) = 36.93, *P* < 0.0001) and a significant interaction between factors (F _(1, 27)_ = 7.455, *P* = 0.0110).

**(E)** PGE2 data for stenotic vs control AVIC. Two-way ANOVA (cell x treatment) revealed a significant main effect of cell (F _(1, 17)_ = 11.61, *P* = 0.0034) and a significant effect of treatment (F _(1, 17)_ = 24.22, *P* = 0.0001) and a significant interaction between factors (F _(1, 17)_ = 5.031, *P* = 0.0385).

**FIGURE 2**

**(D):** Data for stenotic vs control AVIC

- COX2 data. Two-way ANOVA (cell x treatment) revealed a significant main effect of cell (F _(1, 60)_ = 17.00, *P* = 0.0001) and a significant effect of treatment (F _(3, 60)_ = 17.51, *P* < 0.0001) and a significant interaction between factors (F _(3, 60)_ = 3.456, *P* = 0.0219).

- ICAM-1 data. Two-way ANOVA (cell x treatment) revealed a significant main effect of cell (F _(1, 67)_ = 17.40, *P* < 0.0001) and a significant effect of treatment (F _(3, 67)_ = 51.53, *P* < 0.0001) and a significant interaction between factors (F _(3, 67)_ = 7.331, *P* = 0.0003).

**(F)** Data for control AVIC vs control PVIC

- COX2 data. Two-way ANOVA (cell x treatment) revealed a significant main effect of cell (*F _(1, 53_*) = 7.882, *P* = 0.0041) and a significant effect of treatment (F _(3, 48)_ = 12.93, *P* < 0.0001) and a significant interaction between factors (F _(3, 53_) = 3.668, *P* = 0.0178).

- ICAM-1 data. Two-way ANOVA (cell x treatment) revealed a significant main effect of cell (*F _(1, 48_*) = 9.075, *P* = 0.0070) and a significant effect of treatment (F _(3, 48)_ = 17.18, *P* < 0.0001) and a non significant interaction between factors (F _(3, 48_) = 2.627, *P* = 0.0610).

**FIGURE 3**

**(A)** PGE2 kinetics data.

- control AVIC. Two-way ANOVA (treatment x time) revealed a significant main effect of treatment (*F _(5, 53_*) = 3.043, *P* = 0.0173) and a non significant effect of time (F _(2, 53)_ = 2.036, P = 0.1406) and a non significant interaction between factors (F _(10, 53_) = 0.8447, *P* = 0.5887).

- stenotic AVIC. Two-way ANOVA (treatment x time) revealed a significant main effect of treatment (*F _(5, 50_*) = 11.72, *P* < 0.0001) and a significant effect of time (F _(2, 50)_ = 9.906, P = 0.0002) and a non significant interaction between factors (F _(10, 50_) = 0.8537, *P* = 0.5809).

**(B)** PGE2 data for stenotic vs control AVIC. Two-way ANOVA (cell x treatment) revealed a significant main effect of cell (F _(1, 36)_ = 64.22, *P* < 0.0001) and a significant effect of treatment (F _(6, 36)_ = 15.89, P < 0.0001) and a significant interaction between factors (F _(5, 36)_ = 4.706, *P* = 0.0021).

**(C)** IL-6 data. Two-way ANOVA (cell x treatment) revealed a significant main effect of cell (F _(1, 24)_ = 67.85, *P* < 0.0001) and a significant effect of treatment (F _(3, 24)_ = 24.20, *P* < 0.0001) and a significant interaction between factors (F _(3, 24)_ = 5.368, *P* = 0.0057).

**(D)** VEGF data. Two-way ANOVA (cell x treatment) revealed a significant main effect of cell (F_(3, 47)_ = 2.983, *P* = 0.0406) and a significant effect of treatment (F _(3, 47)_ = 5.659, *P* = 0.0215) and a non significant interaction between factors (F_(3, 47)_ = 1.361, *P* = 0.2663).

**(E)** sICAM data. Two-way ANOVA (cell x treatment) revealed a significant main effect of cell (F_(1,63)_ = 18,73, *P* < 0.0001) and a significant effect of treatment (F _(5, 63)_ = 26.08, P < 0.0001) and a significant interaction between factors (F_(5, 63)_ = 4.728, *P* = 0. 0.0010).

**FIGURE 4**

**(A)** S1P receptor antagonist data.

- COX-2 data. One-way ANOVA (treatment) revealed a significant main effect of treatment (F_(4,49)_ = 10.05, *P* < 0.0001).

- ICAM-1 data. One-way ANOVA (treatment) revealed a significant main effect of treatment (F_(4,46)_ = 15.65, *P* < 0.0001.

**(C)** sICAM-1 data. One-way ANOVA (treatment) revealed a significant main effect of treatment (F_(4,46)_ = 12. 35, *P* < 0.0001).

**FIGURE 5**

**(B)** Signaling cascade activation in stenotic AVIC.

- p-p38. Two-way ANOVA (treatment x time) revealed a significant main effect of treatment (F_(3,54)_ = 38.11, *P* < 0.0001) and a significant effect of time (F _(2, 54)_ = 4.781, *P* = 0.0123) and a non significant interaction between factors (F_(6, 54)_ = 1.196, *P* = 0.3226).

- p-NF-κB. Two-way ANOVA (treatment x time) revealed a significant main effect of treatment (F_(3,47)_ = 11.19, *P* < 0.0001) and a significant effect of time (F _(2, 47)_ = 4.774, *P* = 0.0130) and a non significant interaction between factors (F_(6, 47)_ = 1.333, *P* = 0.02616).

- pERK. Two-way ANOVA (treatment x time) revealed a significant main effect of treatment (F_(3,56)_ = 13.07, *P* < 0.0001) and a significant effect of time (F _(2, 56)_ = 10.27, *P* = 0.0002) and a non significant interaction between factors (F_(6, 56)_ = 1.362, *P* = 0.2458).

- pJNK. Two-way ANOVA (treatment x time) revealed a significant main effect of treatment (F_(3,61)_ = 3,102, *P* = 0.0331) and a non significant effect of time (F _(2, 61)_ = 1.334, *P* = 0.2710) and a non significant interaction between factors (F_(6, 61)_ = 0.3653, *P* = 0.8981).

**(D)** p-p38 induction in stenotic and control AVIC and PVIC. For the three-way ANOVA analysis, Statgraphics Centurion XVI 16.2.04 software (StatPoint Technologies, Inc. USA; Warrenton, VA) was used. First, to detect outliers, data were analyzed with a Boxplot by using Microsoft Excel 2010. Next, a Levene´s test revealed that variances were not comparable by using Statgraphics Centurion XVI 16.2.04. Therefore, a Box-Cox transformation was performed, and later confirmed with a Levene´s test that variances were comparable. Then, transformed data was used for a three-way ANOVA, by evaluating the effect of the following factors and their corresponding levels:

**Factors and levels**

|  | Factors | | |
| --- | --- | --- | --- |
|  | Cell (C) | Time (t) | Treatment(A) |
| Levels | Control AVIC | 10 min | L |
|  | Stenotic AVIC | 30 min | S1P |
|  | PVIC | 60 min | L+S |

The following table summarizes data from the Three-way ANOVA analysis:

**Three-way ANOVA Type** III

| *Source* | *SS* | *df* | *MS* | *F* | *P* |
| --- | --- | --- | --- | --- | --- |
| A | 0,61392 | 2 | 0,30696 | 9,60 | **0,0001** |
| t | 0,132005 | 2 | 0,0660025 | 2,06 | 0,1310 |
| C | 0,426243 | 1 | 0,426243 | 13,33 | **0,0004** |
| At | 0,173661 | 4 | 0,0434153 | 1,36 | 0,2520 |
| AC | 0,215979 | 2 | 0,107989 | 3,38 | **0,0371** |
| tC | 0,0533512 | 2 | 0,0266756 | 0,83 | 0,4364 |
| ABC | 0,0747104 | 4 | 0,0186776 | 0,58 | 0,6746 |
| Residual | 4,21996 | 132 | 0,0319694 |  |  |
| TOTAL | 5,9469 | 149 |  |  |  |

Three-way ANOVA (cell x treatment) revealed a significant main effect of cell (*F _(1, 132_*) = 13.33, *P* = 0.0004) and a significant effect of treatment (F _(2, 132)_ = 9.60 P = 0.0001) and a significant interaction between treatment and cell factors (F _(2, 132_) = 3.38 *P* = 0.0371).

For a pairwise comparison, and given that the effect of the time factor was not significant, a Two-way ANOVA with a LSD Fisher posthoc test was performed with GraphPad 6 Prism (San Diego, CA) by analyzing the factors cell and treatment, at the time of 10 min (maximal p-p38 induction). Two-way ANOVA (cell x treatment) revealed a significant main effect of cell (*F _(2, 44_*) = 29.25, *P* < 0,0001) and a significant effect of treatment (F _(2, 44)_ = 13.44, *P* < 0,0001) and a non-significant interaction between treatment and cell factors (F _(4, 44_) = 1,767, *P* = 0.1539).

**FIGURE 6**

**(B)** Inhibition of signaling routes.

- COX2 data. One-way ANOVA (treatment) revealed a significant main effect of treatment (F_(6,45)_ = 11,49, *P* < 0.0001).

- ICAM-1 data. One-way ANOVA (treatment) revealed a significant main effect of treatment (F_(5,36)_ = 13.42, *P* < 0.0001).

**(C)** sICAM-1 data. One-way ANOVA (treatment) revealed a significant main effect of treatment (F_(4,37)_ = 25.32, *P* < 0.0001).

**FIGURE 7**

**(A)** BMP2 data. Two-way ANOVA (cell x treatment) revealed a significant main effect of cell (F_(1,45)_ = 23.15, *P* < 0.0001) and a significant effect of treatment (F_(3, 45)_ = 20.88, P < 0.0001) and a significant interaction between factors (F_(3, 45)_ = 3.957, *P* = 0.0138).

**(B)** Ca2+ deposition data. Two-way ANOVA (cell x treatment) revealed a significant main effect of cell (F_(1,96)_ = 14.41, *P* = 0.0003) and a significant effect of treatment (F _(4, 96)_ = 16.58, *P* < 0.0001) and a non significant interaction between factors (F_(4, 96)_ = 1.299, *P* = 0.2759).

**(D)** ALP data. Two-way ANOVA (cell x treatment) revealed a significant main effect of cell (F_(1, 84)_ = 26.80, *P* < 0.0001) and a significant effect of treatment (F_(4, 84)_ = 9.346, *P* < 0.0001) and a significant interaction between factors (F_(4, 84)_ = 2.892, *P* = 0.0270).

**(E)** ALP data. Two-way ANOVA (cell x treatment) revealed a significant main effect of cell (F_(1,63)_ = 30.72, *P* < 0.0001) and a significant effect of treatment (F _(4, 63)_ = 30.11, *P* < 0.0001) and a significant interaction between factors (F_(4, 63)_ = 6.291, *P* = 0.0003).

**(F)** One-way ANOVA (treatment) revealed a significant main effect of treatment (F_(5,36)_ = 6.942, *P* = 0.0001).

**(G)** One-way ANOVA (treatment) revealed a significant main effect of treatment (F_(4,19)_ = 11,35, *P* < 0.0001).
